# A New Mechanism for the Inhibition of SA106 Gr.B Carbon Steel Corrosion by Nitrite in Alkaline Water

**DOI:** 10.3390/ma17184470

**Published:** 2024-09-12

**Authors:** Do-Haeng Hur, Jeoh Han, Joung-Hae Lee, Soon-Hyeok Jeon, Hee-Sang Shim

**Affiliations:** 1 Materials Safety Technology Development Division, Korea Atomic Energy Research Institute, Daejeon 34057, Republic of Korea; johan2965@khnp.co.kr (J.H.); junsoon@kaeri.re.kr (S.-H.J.); hshim@kaeri.re.kr (H.-S.S.); 2 S∙FAC, Ltd., Daejeon 34025, Republic of Korea; jhlee@s-fac.co.kr

**Keywords:** carbon steel, nitrite, corrosion inhibition, polarization, X-ray photoelectron spectroscopy, passive film

## Abstract

The purpose of this study was to investigate the composition of oxide films formed on SA106 Gr.B carbon steel in nitrite solutions at 35 °C for 1000 h. The product of the reduction of nitrite during the corrosion inhibition process was also examined. The X-ray photoelectron spectroscopy results revealed that a thin Fe_3_O_4_ film was formed and ammonium ions were adsorbed on the outermost surface of the oxide film. The presence of ammonium ions was also demonstrated by ion chromatography. These results indicate that nitrites are reduced to ammonium ions, which in turn promotes the formation of the protective Fe_3_O_4_ film.

## 1. Introduction

Carbon steel is widely used in rebars embedded in concrete, pipe materials used to transport liquids and gas, and various other construction materials. However, carbon steel is susceptible to general corrosion, pitting, and flow-accelerated corrosion [1,2,3]. To address this, its corrosion can be controlled by adding inorganic inhibitors including nitrite [4,5], chromate [6,7], molybdate [8,9], tungstate [10,11], phosphate [12,13], hydrazine [14,15], or their mixtures [7,10,11]. Among them, nitrite (NO_2_^−^) has attracted attention because of its low cost and good inhibition performance.

It has been proposed that nitrite acts as an oxidizing agent and promotes the formation of a robust and stable passive film of iron oxides on the carbon steel’s surface [4,16]. Polarization curves of the carbon steel show that the addition of nitrite shifts the corrosion potential to more noble values and decreases the corrosion current density, confirming that nitrite is an oxidizing inhibitor [4,5,17]. Notably, the oxide films that form in the presence of nitrite are less porous, more uniform, and more compact compared to those formed without nitrite [18,19]. Furthermore, nitrite decreases the donor density in passive films, thereby enhancing the stability of the films [20,21]. Its inhibition capability has also been demonstrated in solutions containing chloride, although in this case higher nitrite concentrations are needed to prevent corrosion [22,23,24]. In addition, nitrite effectively promotes the growth of a passive oxide layer on copper and improves surface homogeneity [25].

However, there is still no consensus about the composition of the iron oxide films formed on carbon steel during the inhibition process caused by nitrite. The most popular oxide film candidate is γ-Fe_2_O_3_ (maghemite) [17,21,22,26,27,28,29,30,31]. The presence of γ-Fe_2_O_3_ has mainly been supported by Fourier transform infrared spectroscopy. In addition, several other different types of oxides have been reported to form: Fe_2_O_3_ [32,33], Fe(OH)_3_ [34], FeOOH [23]. All of these oxides have a single composition. However, some researchers have reported mixed iron oxides with different compositions: Fe_3_O_4_-γ-Fe_2_O_3_ [35], FeO/Fe_2_O_3_/FeOOH [36,37], FeOOH/Fe_2_O_3_ [38], FeO/Fe_3_O_4_ [39].

There are also conflicting claims about the nitrite reduction products formed during the corrosion inhibition process. Many researchers have suggested that NO_2_^−^ ions are reduced to NO gas [17,21,22,26,27,28,29,31,32,40,41]. Various other substances have also been proposed as reduction products of nitrite: NH_3_ (g) [34,35], N_2_ (g) [30], N_2_O (g) [17,23,26,42], NH_4_^+^ [28,33,43]. However, it should be noted that, presently, only suggestions have been made about the creation of these substances, and their existence has not been experimentally proven so far.

In this study, we examined oxide films formed on SA106 Gr.B carbon steel in simulated alkaline closed cooling water with and without nitrite using X-ray photoelectron spectroscopy (XPS) and X-ray diffraction (XRD) methods. The product of the reduction of nitrite was also investigated using ion chromatography. Based on the results, a new corrosion inhibition mechanism is presented.

## 2. Materials and Methods

### 2.1. Specimen and Solution Preparation

Specimens were manufactured from an SA106 Gr.B carbon steel pipe (Sumitomo Metals, Wakayama, Japan) using wire-cutting electrical discharge machining. Their dimensions were 5 mm (width) × 10 mm (length) × 1.5 mm (thickness) for the polarization tests, and 20 mm (width) × 25 mm (length) × 1.5 mm (thickness) for the immersion tests. The chemical composition of the carbon steel is given in Table 1. The surfaces of the specimens were ground using silicon carbide paper, finished with a grit size of #1200, and then ultrasonically cleaned in acetone.

Solutions for the polarization and immersion tests were prepared using deionized water and analytical-grade sodium nitrite. The concentrations of nitrite in each solution were 0, 50, and 600 ppm in weight. The pH of all the solutions was adjusted to 10.0 ± 0.1 with ethanolamine to exclude the effect of pH on the corrosion behavior of the steel. The amount of ethanolamine required to adjust the pH_25°C_ of demineralized water to 10 was 26 mg/kg, when calculated using the MULTEQ code (Version 4.2.0). However, in this study, the pH values of the test solutions were adjusted by adding diluted ethanolamine to the solutions until the pH meter readings reached the target value of 10.

The test solutions were not de-aerated. The temperature of the solutions was maintained at 35 °C using a heating mantle during the tests. These test conditions were chosen to simulate the closed cooling water conditions of nuclear power plants [44]. The normal nitrite concentration range for plant operation is 500–1500 ppm in the form of NO_2_^−^ [44]. Nitrite exposure has been shown to adversely affect humans and ecosystems [45]. Therefore, the nitrite concentration of 50 ppm was tested to explore the possibility of lowering the nitrite concentration.

### 2.2. Potentiodynamic Polarization Test

Potentiodynamic polarization tests were performed in a conventional three-electrode glass cell filled with 650 mL of test solution using a Gamry Reference 600 potentiostat (Gamry Instruments, Warminster, PA, USA). A saturated calomel electrode (SCE) was used as a reference electrode and a coiled platinum wire was used as a counter electrode. A surface-finished specimen was spot-welded to a pure iron lead wire and then the wire was electrically insulated with a heat-shrinkable polytetrafluoroethylene tube. Resin was applied around the welded point. This specimen was used as a working electrode and its surface area was approximately 1.3 cm^2^. After the open circuit potential of the working electrode reached a stable value in each test solution, the potential was scanned from −0.3 V versus the open circuit potential to 1.1 V_SCE_ in the positive direction at a sweep rate of 30 mV/min. The tests were carried out at least three times to check the reproducibility of the results using a new specimen and solution.

The inhibition efficiency (*η*) was also calculated using the following equation:

(1) $$\eta (\%)=\frac{i_{0}-i}{i_{0}}$$

where *i*_0_ and *i* are the corrosion current density in the solution without and with the nitrite inhibitor, respectively.

### 2.3. Immersion Corrosion Test

Three corrosion coupons per experimental condition were loaded onto a Teflon holder in a glass corrosion cell. According to the NACE/ASTM standard [46], the preferred minimum ratio of the test solution volume to coupon surface area is 20 mL/cm^2^. In this work, the cell was filled with the solution so that the ratio was 25 mL/cm^2^. The coupons were removed from the corrosion cell to measure the weight change after 24, 72, 500, and 1000 h, respectively, rinsed in deionized water and acetone, and dried with compressed air. The weights of the coupons were measured using an analytical balance with a readability of 10 mg. After the measurement, the coupons were reloaded into the cell filled with fresh solution.

### 2.4. Surface Characterization

The outer surfaces of the coupons after the 1000 h immersion corrosion test were observed using an optical microscope and a scanning electron microscope (SEM). X-ray photoelectron spectroscopy (XPS) was used to examine the chemical compositions and oxidation states of the oxide films formed on the coupon surface. High-resolution photoelectron spectra were collected using a monochromated Al Kα radiation source at a pass energy of 50 eV. The oxide surface was etched using an argon ion beam at an ion energy of 1.0 kV. The binding energy scale of the XPS spectra was calibrated using the reference C 1s peak at 284.6 eV. The background was subtracted from the calibrated spectra using the Shirley method. The spectra were then deconvoluted and interpreted by cross-referencing them against the NIST (National Institute of Standards and Technology) XPS Database Version 5.0 [47] and Handbooks of Monochromatic XPS Spectra [48].

The corrosion products formed on the coupon surfaces in the solution without nitrite were analyzed using an X-ray diffractometer with a copper Kα radiation source (λ = 1.5406 Å). XRD patterns were acquired in the 2θ range from 20° to 80° with a scan rate of 5°/min.

### 2.5. Chromatographic Conditions

Ion chromatography was utilized to quantify NH_4_^+^ concentration in the 600 ppm nitrite solution after the 1000 h immersion corrosion test. A Thermo Fisher Scientific & ICS-6000 system (Thermo Fisher Scientific Inc., Waltham, MA, USA) equipped with Dionex IonPac CG18 guard column and Dionex IonPac CS18 separation column was used to separate cations. Methanesulfonic acid was used as an eluent. The conductivity of the eluent was suppressed using a Cation Dynamically Regenerated Suppressor 600 while increasing the conductivity of the sample ions. The eluent flow rate was set at 0.3 mL/min with an injection volume of 5 μL.

The sample solution was diluted to 1/100 by weight. The NH_4_^+^ standard solutions used to create the calibration curve were prepared using a 1000 mg/kg certified standard solution (CRM number: 105-04-101) manufactured by the Korea Research Institute of Standards and Science (Daejeon, Republic of Korea). Three types of NH_4_^+^ standard solutions (3 μg/kg, 5 μg/kg, 7 μg/kg) were prepared using the gravimetric method to create the calibration curve. The deionized water was used for dilution and the preparation of standard solutions only after confirming ASTM Type 1 specifications. The concentration of NH_4_^+^ in the sample solution was quantified by comparing peak areas in the ion chromatograms of the sample with those of known standards. All measurements were conducted at 25 °C.

## 3. Results and Discussion

### 3.1. Polarization Behavior

**Figure 1:**
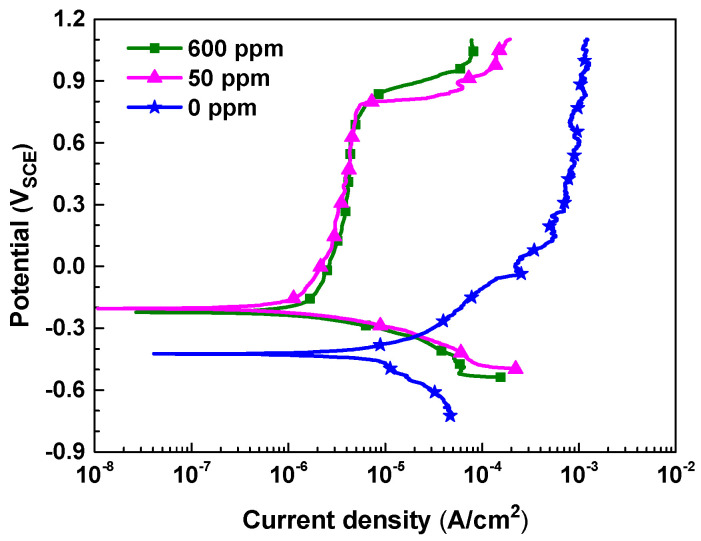
Potentiodynamic polarization curves of SA106 Gr.B carbon steel in the solutions containing 0, 50 and 600 ppm nitrite at 35 °C.

Figure 1 shows the potentiodynamic polarization curves of SA106 Gr.B carbon steel in the test solutions with different nitrite concentrations. The electrochemical corrosion parameters and inhibition efficiency that were determined from the curves are listed in Table 2. In the blank solution (0 ppm nitrite), the corrosion potential was relatively low, and as the polarization progressed above the corrosion potential, active dissolution without passivation was observed, resulting in high anodic current density values. However, in the solutions containing nitrite, the corrosion potential increased and spontaneous passivation occurred, resulting in a significant decrease in the anodic current densities. This result indicates that nitrite acts as an oxidizing agent to promote the oxidation reaction, forming a passive film on the steel surface. In particular, the change in the cathodic curve due to the addition of nitrite was minimal, while the anodic current density values decreased to a few hundredths, confirming that nitrite is an anodic inhibitor. Meanwhile, when the amount of added nitrite was increased from 50 ppm to 600 ppm, no significant change was observed in the corrosion current density value. This suggests that even a small amount of nitrite, 50 ppm, has a corrosion inhibition effect similar to that of 600 ppm nitrite.

### 3.2. Immersion Corrosion Behavior

**Figure 2:**
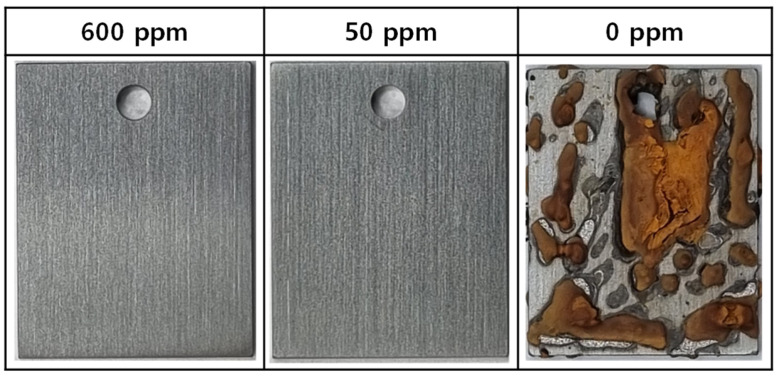
Optical microscope images of the SA106 Gr.B carbon steel surfaces after immersion tests in solutions containing 0, 50, and 600 ppm nitrite at 35 °C for 1000 h.

Figure 2 shows photographs of the surfaces of the corrosion coupons immersed in the test solutions at 35 °C for 1000 h. Under the conditions of 50 ppm and 600 ppm nitrite, there were no signs of corrosion. However, in the blank solution (0 ppm nitrite), it can be seen that severe corrosion occurred over almost the entire surface of the coupons, forming a thick oxide. The oxide formed under this condition was not firmly adhered to the coupon surface and easily crumbled. The outermost layer of the oxide was a dark orange color, while the inner part was black.

**Figure 3:**
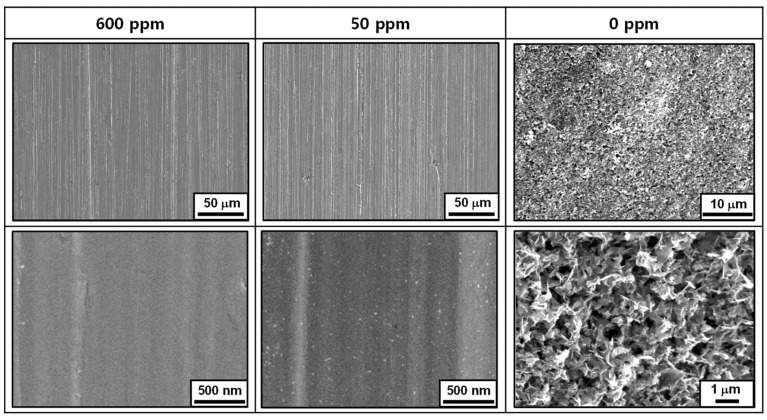
SEM images of the SA106 Gr.B carbon steel surfaces after immersion tests in solutions containing 0, 50, and 600 ppm nitrite at 35 °C for 1000 h.

Figure 3 shows the SEM photographs of the coupons that were immersed in the test solutions at 35 °C for 1000 h. In the solutions containing 50 ppm and 600 ppm nitrite, the coupons still retained the grinding marks caused by the SiC paper during the specimen preparation process, and traces of oxide were only observed at a high magnification of 20,000 times. This suggests that the thickness of the oxide film is extremely thin. However, without added nitrite (0 ppm nitrite), a thick porous oxide was observed.

**Figure 4:**
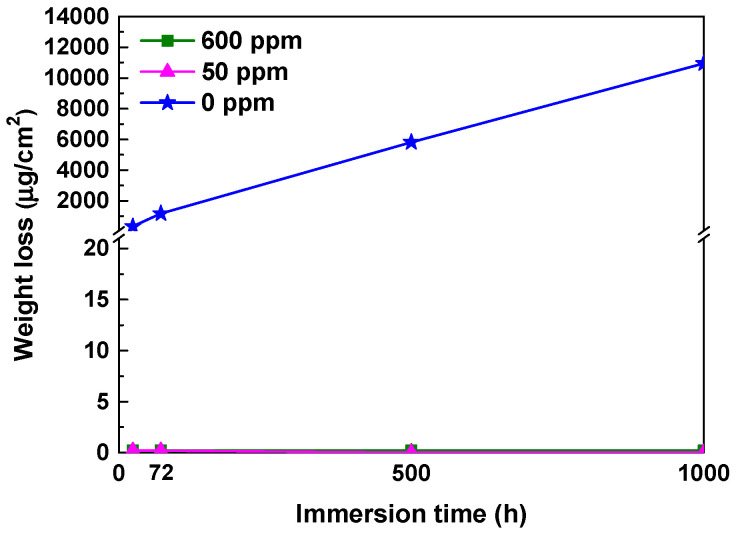
Weight loss of SA106 Gr.B carbon steel during the immersion tests in the solutions containing 0, 50, and 600 ppm nitrite at 35 °C.

Figure 4 illustrates the corrosion behavior based on the weight lost from the coupons. As mentioned earlier, the thick oxides formed in the blank solution (0 ppm nitrite) were not dense and had no adhesion to the coupon surface, making them easily removable by ultrasonic cleaning. Therefore, in this case, weight loss was evaluated after removing the thick oxides with ultrasound. In the absence of nitrite, the amount of weight loss increased almost linearly over the testing time. However, in the solutions containing 50 and 600 ppm nitrite, almost no weight loss occurred. Based on the weight loss and 1000 h test time, the corrosion rate was calculated to be 10.94 μg/cm^2^ h for the blank solution and 0.0004 μg/cm^2^ h for both the 50 and 600 ppm nitrite solutions. The results of the immersion corrosion experiments are in good agreement with the polarization behavior and surface conditions presented in Figure 1 and Figure 2. From the above results, it can be inferred that the addition of nitrite forms a protective film on the specimen’s surface, inhibiting corrosion.

Meanwhile, the electrochemical and immersion corrosion behaviors of carbon steel were nearly the same in the 50 ppm and 600 ppm nitrite solutions. This may indicate that the lower limit of 500 ppm nitrite for the closed cooling water of nuclear power plants, specified by the guidelines [44], is overestimated. However, our results were obtained in stagnant water conditions. Corrosion rates will be affected by the presence and absence of water flow at the same nitrite concentration. Therefore, in order to apply to the closed cooling water system with high flow velocities, the effect of flow velocities on carbon steel corrosion should be considered.

### 3.3. Oxide Characteristics

**Figure 5:**
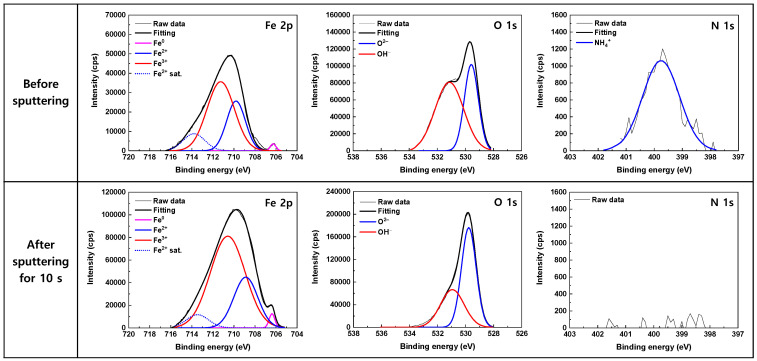
High resolution XPS spectra of Fe 2p, O 1s, and N 1s core-levels for the oxide films formed on SA106 Gr.B in the 600 ppm nitrite solution at 35 °C for 1000 h.

**Figure 6:**
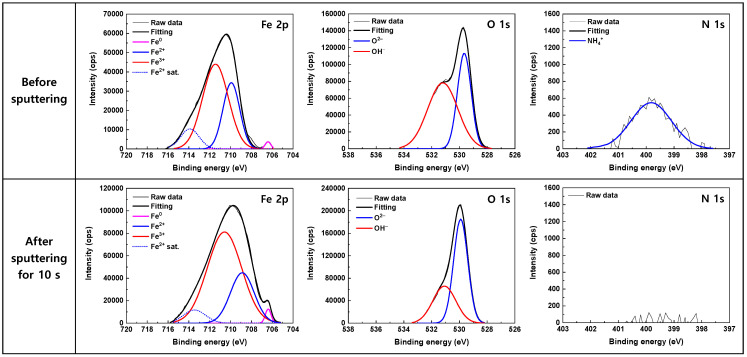
High resolution XPS spectra of Fe 2p, O 1s, and N 1s core-levels for the oxide films formed on SA106 Gr.B in the 50 ppm nitrite solution at 35 °C for 1000 h.

Figure 5 and Figure 6 shows the high-resolution XPS spectra obtained from the surfaces of the corrosion coupons after the 1000 h immersion tests in the 600 ppm and 50 ppm nitrite solutions, respectively. Quite similar results were observed in both the solutions. The corresponding peak parameters of the deconvoluted XPS spectra are summarized in Table 3 The Fe 2p peak was decomposed into Fe^0^, Fe^2+^, and Fe^3+^ peaks. A satellite peak of Fe^2+^ was also observed at a binding energy of 714 eV. The information in the XPS peak intensity comes from the outermost 5–10 nm of the sample [49]. Therefore, the detection of metallic Fe^0^ at 706 eV suggests that the thickness of the oxide film is less than 5–10 nm. Fe^2+^ ions at 709 eV may exist in the forms of FeO, Fe(OH)_2_, and Fe_3_O_4_, while Fe^3+^ ions at 711 eV may exist in the forms of Fe_2_O_3_, Fe_3_O_4_, and FeOOH, making it very difficult to determine the iron oxide composition present on the surface. The asymmetric O 1s peak consisted of O^2–^ and OH^−^ components. The O^2–^ peak at 529.5 eV corresponds to the iron oxide lattice, while the OH^−^ peak at 531.1 eV is attributed to iron hydroxide. Therefore, it can be inferred that both Fe(II)/Fe(III) oxide and hydroxide coexist on the specimen surface. As the OH^−^ peak significantly decreases after 10 s of sputtering, Fe(II) hydroxide appears to be mainly distributed in the outermost layer.

In Table 3, the ratio of the Fe^3+^ to Fe^2+^ peak area was 1.96–2.06 on the as-received surface before sputtering, indicating that the stoichiometry of the oxide is closely analogous to that of Fe_3_O_4_. After 10 s of sputtering, the ratio of the Fe^3+^ to Fe^2+^ peak area slightly increased to 2.31–2.62. This may be due to the removal of the Fe(II) hydroxide in the outermost layer during the sputtering.

An N 1s peak was also detected at a binding energy of 399.8 eV as shown in Figure 5 and Figure 6. The peak showed a Gaussian distribution with a FWHM of 1.54 eV, indicating that it consisted of a single species. Notably, the intensity of the peak was reduced to approximately half when the nitrite concentration decreased from 600 ppm to 50 ppm. However, after 10 s of sputtering, the N peak was no longer observed, while the Fe 2p and O 1s peaks were still detected with high intensities. The binding energies of several N-species have been reported as follows: 396.9–399.0 eV for atomic N [50,51,52,53], 400.1–401.0 eV for NH_4_^+^ [54,55,56,57], 401.3–403.5 eV for NO [50,58,59,60], 403.5–404.5 eV for NO_2_^−^ [61,62,63,64], 405.4–406.0 eV for physisorbed NO_2_ [53,65,66], 407.1–408.1 eV for NO_3_^−^ [61,62,63,64]. It can be seen that the binding energies of NH_4_^+^ and NO are close, even though most of the binding energies of NO have been reported to be in the higher energy region. As a result, it is not easy to determine where the N 1s peak originates. However, the formation of NH_4_^+^ from NO_2_^−^ was confirmed by ion chromatography and thermodynamic calculation, which will be described later. Consequently, it is reasonable that the N 1s peak centered at 399.8 eV is assigned to NH_4_^+^. The thermochemical radius for NH_4_^+^ is 1.36 Å [67]. The sputtering rate for Fe_2_O_3_ under the XPS examination conditions used in this study is estimated to be approximately 6 Å/min [68]. (The sputtering rate for Fe_3_O_4_ is not available in the literature.) In other words, assuming that the oxide film consists of Fe_2_O_3_, the film layer that is approximately 1 Å thick is removed by sputtering for 10 s. Therefore, the results of the N 1s spectra indicate that NH_4_^+^ is just adsorbed on the outermost layer of the oxide film as a single molecular layer and is not incorporated into the film. Until now, most XPS studies related to nitrite inhibitors have only shown the spectra of Fe and O, but have paid little attention to those of N. Among the exceptions, Al-Refaie et al. proposed that the N 1s signal at 400.1 eV may arise from N_2_ [30], while Fujioka et al. assigned the peak at 400.6 eV to NH_4_^+^ [43].

The process of inhibiting corrosion on carbon steel by adding nitrite has been known to occur as nitrite is reduced to NO, forming a protective layer of γ-Fe_2_O_3_ on the carbon steel surface, thereby inhibiting corrosion according to the following reaction [21,22,26,27,69].

2Fe^2+^ + 2OH^−^ + 2NO_2_^−^ → 2NO + γ-Fe_2_O_3_ + H_2_O (2)

The XPS analysis results in Figure 5 and Figure 6 and Table 2 reveal that the surface is covered by a thin layer of iron oxides containing both Fe^2+^ and Fe^3+^ ions. However, γ-Fe_2_O_3_ is an iron oxide composed of only Fe^3+^ ions, and thus differs from the results of this experiment. Furthermore, neither NO_2_^−^ nor NO were detected in the outer layer of the oxide film, and only NH_4_^+^ was consistently detected. This indicates that the added NO_2_^−^ does not adsorb onto the surface, and that the reduction product of NO_2_^−^ is NH_4_^+^, not NO.

HSC Chemistry 6 software (Version 6.12) [70] was used to calculate the Gibbs free energy (ΔG_35°C_) of several reactions at 35 °C. The thermodynamic calculations revealed that NO_2_^−^ can be reduced easily to NH_4_^+^, as follows:

NO_2_^−^ + 8H^+^ + 6e^−^ → NH_4_^+^ + 2H_2_O     (ΔG_35°C_ = −519.0 kJ/mol) (3)

The formation reactions of various iron oxides can be expressed by the following equations.

3Fe + 4H_2_O → Fe_3_O_4_ + 4H_2_     (ΔG_35°C_ = −66.8 kJ/mol) (4)

2Fe + 3H_2_O → Fe_2_O_3_ + 3H_2_     (ΔG_35°C_ = −31.7 kJ/mol) (5)

Fe + 2H_2_O → α-FeOOH + 3/2H_2_     (ΔG_35°C_ = −15.8 kJ/mol) (6)

Fe + 2H_2_O → γ-FeOOH + 3/2H_2_     (ΔG_35°C_ = −6.6 kJ/mol) (7)

Fe + 3H_2_O → Fe(OH)_3_ + 3/2H_2_     (ΔG_35°C_ = 5.2 kJ/mol) (8)

The thermodynamic calculations indicate that the formation of Fe_3_O_4_ is favored in the test conditions used in this work. The ratio of Fe^3+^ to Fe^2+^ in pure magnetite is 2:1, which is consistent with the XPS results. Therefore, it is proposed that NO_2_^−^ is electrochemically reduced to NH_4_^+^ on the surface and Fe is simultaneously oxidized to form iron oxides (i.e., Fe_3_O_4_) containing Fe^2+^ and Fe^3+^ ions.

In addition, Fe_3_O_4_ can also be formed through the following reactions.

Fe + 2H_2_O → Fe(OH)_2_ + H_2_     (ΔG_35°C_ = −18.2 kJ/mol) (9)

3Fe(OH)_2_ → Fe_3_O_4_ + 2H_2_O + H_2_     (ΔG_35°C_ = −12.3 kJ/mol) (10)

The ΔG_35°C_ for all the above equations has a negative value. Fe_3_O_4_ is generally known to be formed through the two-step reaction process (Schikorr reaction) shown in Equations (9) and (10). These reactions are likely to be related to the Fe(II) hydroxide detected in the XPS spectra in Figure 5 and Figure 6. Nevertheless, based on the ΔG_35°C_ values, the one-step reaction presented in Equation (4) is thermodynamically preferred under these experimental conditions.

Based on the experimental results and thermodynamic calculations, the mechanism of corrosion inhibition with the addition of nitrite is proposed to follow the overall reaction equation below.

3Fe + NO_2_^−^ + 2H_2_O + 8H^+^ + 6e^−^ → Fe_3_O_4_ + NH_4_^+^ + 4H_2_ (11)

The ΔG_35°C_ for Equation (11) is −585.8 kJ/mol, demonstrating that the reaction occurs spontaneously. In summary, the reduction reaction of nitrite promotes the formation of a protective Fe_3_O_4_ film on the carbon steel surface, thereby inhibiting material corrosion. In this process, the added NO_2_^−^ is not adsorbed onto the steel’s surface; instead, its reduction product, NH_4_^+^, is adsorbed onto the outermost layer. In alkaline aqueous solutions, the zeta potential of oxide films and material surfaces has a negative value [71,72,73,74,75]. Therefore, the adsorption of NO_2_^−^ is not favored because a repulsive force acts between both the negatively charged NO_2_^−^ and the steel surface. Conversely, since an attractive force acts between the positively charged NH_4_^+^ and the negatively charged steel surface, the adsorption of NH_4_^+^ is facilitated. As a result, the detection of NH_4_^+^ on the outermost layer can also be explained in terms of this zeta potential.

In the Section 1, we noted that different researchers have reported that a wide variety of iron oxides are formed on carbon steel in nitrite solutions. This is not a matter of what is right or wrong, but should be viewed as a reflection of the oxidizing power of the test solutions depending on the experimental conditions. In other words, the driving force for the oxidation of the steel will depend on the nitrite concentration, type of nitrite salt, pH, temperature, the presence or absence of chloride, etc. The reduction kinetics of nitrite to ammonium ion would also be affected by these factors. As a result, in an environment where the redox potential is sufficiently high, only trivalent iron oxides will be formed; otherwise, a mixture of divalent and trivalent iron oxides will be formed.

**Figure 7:**
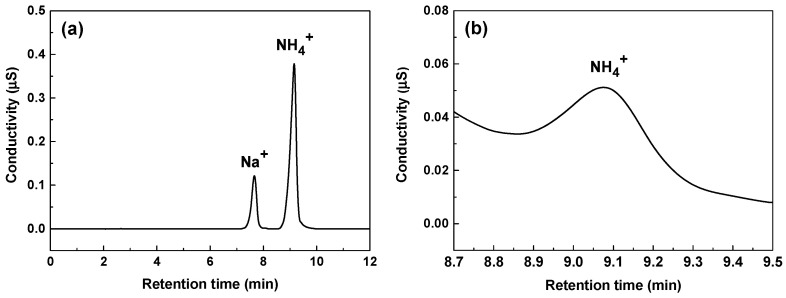
Chromatograms: (**a**) mixed standard solution containing 10 mg/L ammonium and 2 mg/L sodium ions, and (**b**) sample solution from the 600 ppm nitrite solution after the immersion test at 35 °C for 1000 h.

To verify the presence of NH_4_^+^, ion chromatography analysis was conducted on the 600 ppm nitrite solution following the 1000 h immersion corrosion test. Na^+^ and NH_4_^+^ are known to elute in close proximity to each other [76,77], and thus the as-received sample itself could not be analyzed directly. To overcome this problem, we diluted the sample solution to 1/100 by weight, and used methanesulfonic acid as an eluent and a Cation Dynamically Regenerated Suppressor. This greatly increased the resolution between Na^+^ and NH_4_^+^ as shown in Figure 7a. The retention times of Na^+^ and NH_4_^+^ were 7.64 and 9.07 min, respectively, which allowed the NH_4_^+^ concentration to be determined without interference from Na^+^. As shown in Figure 7b, a symmetric NH_4_^+^ peak at a retention time of 9.07 min was consistently observed in the sample solution. The NH_4_^+^ concentration calculated using the chromatographic area was 479 ± 13 μg/kg. This result not only demonstrates the presence of ammonium ions, but is also consistent with the detection of NH_4_^+^ by XPS. The final pH values of the test solutions were adjusted with ethanolamine as described in the experimental section. Thermolysis of ethanolamine led to the formation of mostly acetate and formate, with some traces of glycolate [78]. Therefore, it is confirmed that the NH_4_^+^ ions originated from NO_2_^−^ ions. The reduction reaction (3) of NO_2_^−^ to NH_4_^+^ was proposed by Pourbaix [79] and, to the authors’ knowledge, was first applied to explain the inhibition action of nitrite by Joseph et al. [28], but they failed to detect ammonium ions.

**Figure 8:**
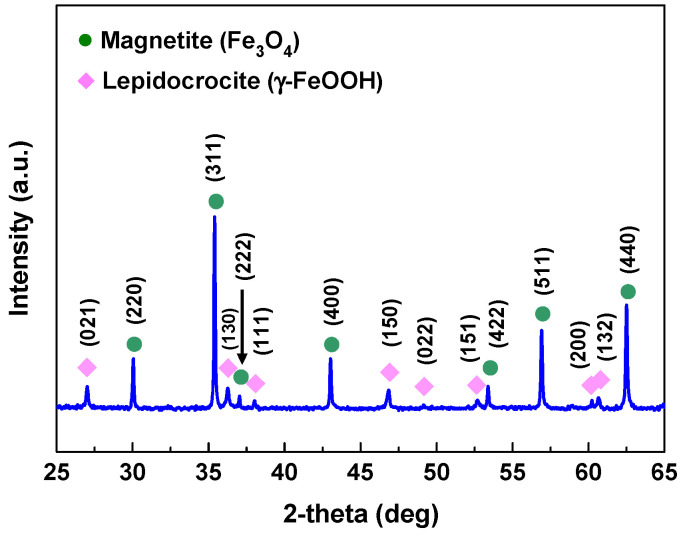
XRD patterns of the oxides formed on SA106 Gr.B in the blank solution (0 ppm nitrite) at 35 °C for 1000 h.

Because thick and easily crumbling oxides were formed in the blank solution (0 ppm nitrite), the oxide particles were scraped off with a plastic knife and subjected to XRD analysis. As shown in Figure 8, the diffraction angles of the oxide precisely matched those of cubic Fe_3_O_4_ (PDF Card No. 01-079-0418) and orthorhombic γ-FeOOH (lepidocrocite, PDF Card No. 01-070-8045). Considering the diffraction intensities and peak areas, Fe_3_O_4_ appears to be the primary component of the oxide. As described in Figure 2, the outermost surface of the oxide is a dark orange color while the inside is black. Therefore, it seems that γ-FeOOH exists in the outermost layer and Fe_3_O_4_ in the inner layer. It can be inferred that γ-FeOOH is produced according to reaction (12) and subsequently γ-FeOOH is transformed to Fe_3_O_4_ according to reaction (13).

4Fe(OH)_2_ + O_2_ → 4γ-FeOOH + 2H_2_O     (ΔG_35°C_ = −461.7 kJ/mol) (12)

2γ-FeOOH + Fe(OH)_2_ → Fe_3_O_4_ + 2H_2_O   (ΔG_35°C_ = −17.0 kJ/mol) (13)

## 4. Conclusions

This study investigated the corrosion behavior of SA106 Gr.B carbon steel in solutions with and without nitrite at 35 °C for 1000 h. In the blank solution, the steel showed a high corrosion rate of 10.94 μg/cm^2^ h, and was covered with thick and easily crumbling oxides composed of Fe_3_O_4_ and γ-FeOOH. With the addition of 50~600 ppm nitrite, the corrosion rate was greatly reduced to 0.0004 μg/cm^2^ h, and an extremely thin Fe_3_O_4_ film of less than 5–10 nm was formed. Ammonium ions were identified on the oxide film surface and in the nitrite solution, and ammonium ions were only adsorbed to the outermost layer and were not incorporated into the oxide film. The nitrite ions were electrochemically reduced to ammonium ions, which provided the driving force for the oxidation of carbon steel into a protective Fe_3_O_4_ film. The overall reaction of corrosion inhibition on carbon steel can be described as follows.

3Fe + NO_2_^−^ + 2H_2_O + 8H^+^ + 6e^−^ → Fe_3_O_4_ + NH_4_^+^ + 4H_2_ (14)

Here, the chemical composition of iron oxide is thought to depend on the oxidizing power of the nitrite solutions and therefore systematic research on this appears to be necessary in the future.

## Figures and Tables

**Table 1:** Chemical composition of SA106 Gr.B (wt. %).

Ni	Cr	Mo	Cu	C	Fe
0.02	0.04	0.01	0.01	0.19	Balance

**Table 2:** Electrochemical corrosion parameters and inhibition efficiency determined from the polarization curves.

Nitrite Concentration (ppm)	E_corr_ (V_SCE_)	i_corr_ (10^−6^ A/cm^2^)	*η* (%)
0	−0.424	7.78	-
50	−0.204	1.39	82
600	−0.223	1.41	82

**Table 3:** Peak parameters of the deconvoluted XPS spectra before and after 10 s of sputtering.

	Component	600 ppm NO_2_^−^			50 ppm NO_2_^−^		
		BE (eV)	FWHM * (eV)	Relative Area (%)	BE (eV)	FWHM (eV)	Relative Area (%)
Before sputtering	Fe^0^ 2p_3/2_	706.27	0.61	1.2	706.40	0.65	1.1
	Fe^2+^ 2p_3/2_	709.84	2.10	29.4	709.93	1.89	30.0
	Fe^3+^ 2p_3/2_	711.33	3.22	60.5	711.45	2.91	58.9
	Fe^2+^ sat. 2p_3/2_	713.97	2.30	8.9	713.90	2.09	10.0
	O^2–^ 1s	529.57	1.17	38.1	529.57	1.17	38.1
	OH^−^ 1s	531.14	2.40	61.9	531.14	2.40	61.9
	NH_4_^+^ 1s	399.76	1.54	100	399.76	1.54	100
After a 10 s sputtering	Fe^0^ 2p_3/2_	706.23	0.63	1.6	706.38	0.64	1.7
	Fe^2+^ 2p_3/2_	708.88	2.91	27.7	708.87	2.68	25.4
	Fe^3+^ 2p_3/2_	710.69	4.24	64.2	710.59	3.89	66.7
	Fe^2+^ sat. 2p_3/2_	714.16	2.64	6.5	713.46	2.50	6.2
	O^2–^ 1s	529.78	1.26	62.0	529.91	1.20	63.3
	OH^−^ 1s	531.00	2.07	38.0	531.08	1.95	36.7
	NH_4_^+^ 1s	N.D. *	-	-	N.D.	-	-

*: N.D.: not detected, FWHM: full width at half maximum.

## Data Availability

The data are not publicly available due to legal or ethical reasons.
